# Rehabilitation-oriented assessment of functional asymmetry and sensorimotor deficits following total knee arthroplasty: implications for therapeutic strategies in musculoskeletal care

**DOI:** 10.3389/fmed.2026.1760680

**Published:** 2026-03-27

**Authors:** Ghada Mohamed Koura, Ahmed Mohamed Fathi Elshiwi, Ravi Shankar Reddy, Zeinab A. Ali, Tahani Miftah Ahmed Al-Essa, Ebtihaj Awwad Mohammed Alahmari, Hajer Ahmed Ali Asiri, Badriyh Mohammed Abdullah Asiri, Ahmed M. El Melhat

**Affiliations:** 1 Program of Physical Therapy, Department of Medical Rehabilitation Sciences, College of Applied Medical Sciences, King Khalid University, Abha, Saudi Arabia; 2 Department of Physical Therapy, Saudi German Hospital, Abha, Saudi Arabia; 3 Department of Physical Therapy and Health Rehabilitation, College of Applied Medical Science, Jouf University, Al-Jouf, Saudi Arabia; 4 Department of Physical Therapy for Surgery, Cairo University, Cairo, Egypt; 5 Department of Physical Therapy, Azm Physiotherapy Center, Abha, Saudi Arabia; 6 Department of Physical Therapy, Step of Hope Center, Abha, Saudi Arabia; 7 Department of Physical Therapy, Ruh Al-Israr Center for Rehabilitation, Abha, Saudi Arabia; 8 Department of Physical Therapy, Faculty of Health Sciences, Beirut Arab University, Beirut, Lebanon; 9 Department of Physical Therapy for Musculoskeletal Disorders and its Surgery, Faculty of Physical Therapy, Cairo University, Cairo, Egypt

**Keywords:** knee arthroplasty, motor activity, patient reported outcome measures, postural balance, rehabilitation

## Abstract

**Background:**

Functional asymmetry and sensorimotor control impairments often persist in patients following total knee arthroplasty (TKA), yet they remain under-evaluated in routine clinical settings using simple, scalable tools. Understanding the relationship between these deficits and postoperative outcomes is critical for optimizing rehabilitation strategies.

**Objectives:**

To assess the prevalence and degree of functional asymmetry in individuals 6–12 months post-TKA using standardized physical therapy assessments, and to evaluate the predictive value of asymmetry and sensorimotor deficits on both functional performance and patient-reported outcomes.

**Methods:**

A cross-sectional study was conducted on 125 patients aged 55–80 years, 6–12 months after unilateral primary TKA. Functional asymmetry was measured using the Single-Leg Sit-to-Stand (SLSST), Step-Down Test, and modified Star Excursion Balance Test (mSEBT). Functional outcomes included the Timed Up and Go (TUG) and 30-Second Chair Stand Test (30CST). In contrast, self-reported outcomes were assessed he KOOS-ADL and the KOOS–Function in Sport and Recreation (KOOS-Sport/Rec) subscales were administered. Correlations and multiple regression models were used to analyze relationships between asymmetry measures and outcomes.

**Results:**

Significant correlations were found between asymmetry measures and both functional and self-reported outcomes (e.g., SLSST asymmetry and TUG: *r* = 0.47, *p* = 0.005; KOOS-ADL: *r* = −0.45, *p* = 0.007). Regression models identified SLSST, Step-Down, and mSEBT asymmetry as significant predictors of TUG (R^2^ = 0.48, *p* < 0.001) and KOOS-ADL (R^2^ = 0.53, *p* < 0.001). Patients with high asymmetry exhibited significantly poorer outcomes across all measures (all *p* < 0.01).

**Conclusion:**

Functional asymmetry and sensorimotor deficits are independently associated with poorer mobility and self-reported function post-TKA and can be effectively identified using clinically accessible tools, supporting their integration into routine postoperative assessment and rehabilitation planning.

## 1 Introduction

Total knee arthroplasty (TKA) is widely accepted as an effective method for reducing pain and restoring function in individuals with end-stage knee osteoarthritis (1). As the population ages and degenerative joint disease becomes more common, the global need for TKA continues to increase, making it one of the most common orthopedic procedures (2). This procedure is primarily indicated for individuals with advanced, end-stage knee osteoarthritis who have not responded to conservative management, and it is widely recognized as one of the most frequently performed elective orthopedic surgeries in this specific clinical context (2). Despite promising long-term implant success, many patients still face ongoing functional limitations, especially within the first year after surgery (3). Common problems include persistent issues with strength, mobility, and balance; approximately 20%–30% of patients are dissatisfied with their functional outcomes (1). These results underscore the importance of prioritizing functional recovery as patients experience it in their daily lives, rather than focusing solely on radiographic and surgical success (4).

Emerging evidence shows that functional asymmetry–especially in unilateral tasks–and deficits in sensorimotor control can impede optimal recovery after TKA (5, 6). Studies have demonstrated asymmetrical limb loading, strength imbalances, and altered neuromuscular control patterns that may persist into the mid-to-late postoperative period (7). John et al. (8) observed that inter-limb strength differences significantly impact mobility outcomes after TKA, while Almonroeder et al. (9) found that these asymmetries often persist at 1 year and are associated with delayed return to function. Although some prior investigations have included mixed or adjacent arthroplasty populations, including total hip arthroplasty, the underlying mechanisms of inter-limb strength imbalance, altered load distribution, and neuromuscular asymmetry are biomechanically comparable and provide relevant insight into persistent functional discrepancies following unilateral joint replacement procedures (9), Wang et al. (10). Additionally, the study also emphasized the importance of postural control and limb coordination in predicting falls and gait inefficiency in TKA patients. However, many of these findings rely on tools such as instrumented gait analysis, motion capture systems, or isokinetic testing, which are not commonly available in routine outpatient rehabilitation settings. As a result, applying this knowledge in clinical practice remains limited.

Although functional asymmetry and sensorimotor control impairments have been linked to delayed or incomplete recovery, their assessment is not routinely included in standard TKA follow-up, especially when practical, feasible tools are used in real-world clinical settings (5, 11). Most postoperative evaluations still depend on bilateral or general performance measures, such as the Timed Up and Go or 30-Second Chair Stand Test, which may miss subtle yet clinically significant inter-limb differences (12–14). Additionally, while patient-reported outcome measures (PROMs) such as the Knee Injury and Osteoarthritis Outcome Score (KOOS) are commonly used, they may not fully capture the motor control deficits underlying subjective functional limitations (15–17). There is a clear need to investigate whether simple, low-resource physical therapy assessments can effectively measure asymmetry and sensorimotor dysfunction–and whether these factors can account for variability in both objective performance and PROMs. Addressing this gap is crucial for improving personalized rehabilitation strategies and identifying patients at risk of prolonged impairment.

This study aimed to examine the relationship between functional asymmetry, sensorimotor control deficits, and postoperative outcomes in individuals 6–12 months after unilateral primary TKA. Specifically, the study sought to (1) measure the extent of asymmetry using standardized clinical tools and evaluate its link with functional performance and PROMs, and (2) determine whether sensorimotor control measures can predict mobility and self-reported function. It was hypothesized that greater asymmetry and poorer sensorimotor control would be significantly linked to slower mobility, reduced strength, and lower patient-reported functional scores.

## 2 Materials and methods

### 2.1 Study design, ethics, and settings

This cross-sectional study was conducted between October 2023 and May 2024 at the Outpatient Rehabilitation Clinic, Department of Medical Rehabilitation Sciences, King Khalid University, Kingdom of Saudi Arabia. Ethical approval was obtained from the institutional review board of King Khalid University (Approval No: ECM#2023-3209), and written informed consent was obtained from all participants before data collection. All procedures adhered to the ethical principles outlined in the Declaration of Helsinki for research involving human participants.

### 2.2 Participants

Participants were recruited through consecutive sampling from patients attending routine follow-up appointments or those referred to rehabilitation services at the university-affiliated clinic’s outpatient department. All participants were 6–12 months post-surgery, with an average postoperative duration of about 8.2 months. Eligibility was determined based on a clinical diagnosis of unilateral primary total knee arthroplasty performed for advanced knee osteoarthritis, confirmed through surgical and medical evaluation records (18). The inclusion criteria included individuals aged 55–80 years who were between 6 and 12 months post-TKA, medically stable, and able to ambulate independently without the use of assistive devices. Participants had to demonstrate sufficient cognitive ability to understand and follow test instructions and complete self-reported outcome measures. Formal cognitive screening with a standardized psychometric instrument was not conducted because all participants were community-dwelling individuals attending routine outpatient follow-up and had no documented cognitive impairment. Cognitive eligibility was evaluated through structured clinical interaction by a licensed physiotherapist, including assessment of orientation to person, place, and time; the ability to follow multi-step verbal instructions during trial movements; and comprehension of sample questionnaire items before formal data collection. Individuals demonstrating confusion, disorientation, or difficulty following instructions were excluded. Exclusion criteria included revision or bilateral TKA, neurologic disorders (e.g., stroke or Parkinson’s disease), severe contralateral lower-limb osteoarthritis, lower-extremity fractures within the previous year, and active vestibular or balance disorders. All participants underwent a standardized baseline screening to confirm eligibility. This process involved verification of surgical history through medical records, evaluation of pain stability and absence of acute postoperative complications (such as infection, joint instability, or uncontrolled swelling), confirmation of independent ambulation for at least 10 m without assistive devices, and observation of safe performance of sit-to-stand and directional turning tasks without physical assistance. Eligibility was confirmed only when all inclusion criteria were satisfied and no exclusion criteria were identified. All clinical tests and self-reported outcome measures were administered during a single standardized 60-min session, with rest intervals between tests to reduce fatigue-related bias. Functional tests were performed in the following fixed order to minimize fatigue and standardize comparisons: TUG, 30CST, SLSST, Step-Down, and mSEBT. Outcome assessors were blinded to group allocation based on asymmetry classification to decrease detection bias. The complete raw dataset used for statistical analysis is provided as “Supplementary Table” to ensure transparency and reproducibility.

### 2.3 Variables

#### 2.3.1 Timed Up and Go (TUG)

The primary outcome variable in this study was functional mobility, assessed with the Timed Up and Go (TUG) test (19). The TUG measures the time (in seconds) it takes for a participant to stand up from a standard-height chair, walk 3 m, turn around, return, and sit down again (20). This test is commonly used in post-total knee arthroplasty rehabilitation to evaluate dynamic balance, lower limb function, and transitional movement performance. Participants were instructed to perform the test at their usual comfortable pace, and the average of two trials was recorded for analysis. Higher TUG times indicate slower mobility and decreased functional performance. The TUG has shown high reliability (ICC > 0.95) and is considered a valid marker for functional recovery and fall risk in older adults and post-arthroplasty populations (21).

#### 2.3.2 Knee Injury and Osteoarthritis Outcome Score (KOOS)

Self-reported functional outcomes were evaluated using two subscales of the KOOS: the ADL subscale and the Function in Sport and Recreation subscale (22). The official Arabic-translated and culturally adapted version of the KOOS, validated for use in Arabic-speaking orthopedic populations, was administered. If participants had difficulty reading, standardized verbal assistance was provided to ensure accurate responses. Each subscale was scored on a 5-point Likert scale and transformed to a 0–100 scale, where higher scores indicate better function. Participants completed the KOOS independently in a quiet room with standard instructions provided by trained staff. Clarification was permitted only regarding item interpretation, without influencing responses. These outcome measures were selected for their relevance to capturing patient-centered perspectives on functional recovery and for their sensitivity to residual impairments during the subacute to chronic post-TKA phases.

#### 2.3.3 30 -Second Chair Stand Test (30CST)

The 30CST is a validated, performance-based assessment used to measure lower limb muscular strength, endurance, and functional capacity, particularly in older adults and post-surgical populations such as those undergoing total TKA (23). During the test, participants are instructed to rise from a standardized chair to a full standing position and return to sitting as many times as possible within 30 s, without using their arms for assistance (23). This test reflects the functional demands of daily activities like standing from a chair or toilet, which are often challenging for individuals with knee joint impairments. The total number of correctly performed repetitions is recorded, with lower scores indicating diminished lower-extremity strength and endurance.

#### 2.3.4 Single-Leg Sit-to-Stand (SLSST)

The SLSST test was used to evaluate unilateral lower-limb functional strength and motor control (24). Participants were instructed to perform as many complete sit-to-stand repetitions as possible within 30 s using only one leg at a time, starting from a standardized chair height of 45 cm without armrests (24). The non-testing leg was kept off the ground and held in front of the body to prevent compensatory contact. The test was first performed with the non-operated limb, followed by the operated limb, with a standardized 60-s rest interval between trials to minimize fatigue (24). Proper form required full knee and hip extension in the upright position and controlled lowering until the participant’s buttocks contacted the chair. A repetition was counted only if the movement was completed with proper alignment and without using the arms or contralateral limb for support. The number of valid repetitions for each limb was recorded, and SLSST asymmetry was calculated as the absolute inter-limb difference in repetitions (i.e., |Non-operated − Operated|). Higher values indicated greater asymmetry and impaired unilateral strength or neuromuscular control. Participants were allowed one practice repetition per leg before timed testing to ensure comprehension of test mechanics. The SLSST asymmetry score was treated as a continuous predictor variable in correlation and regression analyses.

#### 2.3.5 Step-Down Test

The Step-Down Test was used to evaluate unilateral eccentric control, neuromuscular coordination, and limb asymmetry, particularly relevant in individuals recovering from TKA (25). Participants were instructed to perform as many step-down repetitions as possible within 30 s from a standardized 20 cm (8-inch) step, placed on a level, non-slip surface. The test involved starting from full knee and hip extension on the step with one foot, lowering the contralateral heel to lightly touch the floor without transferring weight, then returning to full standing on the step. Trials were performed separately for the operated and non-operated limbs, with a 60-s rest interval between limbs (25). Proper form was defined as controlled lowering without pelvic tilt, contralateral knee collapse, or use of the upper limbs for assistance. Repetitions were counted only if completed with correct form and within the allotted time. Each participant was provided with one practice trial per limb to ensure familiarity with the movement pattern. The total number of valid repetitions for each limb was recorded, and asymmetry was calculated as the absolute difference between the operated and non-operated limbs (|Operated − Non-operated|). Larger values indicated greater functional asymmetry and potential deficits in limb-specific neuromuscular control. The resulting asymmetry scores were treated as continuous predictor variables in all statistical analyses.

#### 2.3.6 modified Star Excursion Balance Test (mSEBT)

The mSEBT was used to assess dynamic balance, postural control, and sensorimotor asymmetry in the anterior direction, which is known for its high reliability and correlation with functional mobility in post-TKA populations (26). Participants performed the test barefoot, maintaining single-leg stance on the test limb while reaching forward with the opposite limb along a standardized measuring grid. Limb dominance was recorded before testing. Each participant completed four practice trials followed by three recorded trials per limb, with 30-s rest intervals between attempts to minimize fatigue. The maximum anterior reach distance from the three trials was measured using a floor-mounted metric tape. Reach distances were normalized to leg length (anterior superior iliac spine to medial malleolus) and expressed as a percentage of limb length (%LL). Asymmetry was calculated as the absolute difference between the operated and non-operated limbs. The resulting asymmetry scores were treated as continuous predictor variables in correlation and regression analyses.

### 2.4 Covariates/confounders

Demographic and clinical variables known to influence postoperative recovery were included as covariates. These included age (in years), sex (male/female), body mass index (BMI, kg/m^2^), and time since surgery (in months). These variables were collected via structured interview and medical record review during initial enrollment. Age and BMI were treated as continuous variables, while sex was coded dichotomously (male = 1, female = 0). Time since surgery was used to adjust for differences in postoperative rehabilitation exposure and natural recovery trajectories.

### 2.5 Data collection instruments and procedures

All functional tests and questionnaires were administered by licensed physiotherapists trained in standardized administration protocols. Prior to data collection, assessors completed a calibration session to ensure consistency in instruction delivery and test timing. A 5-min warm-up (stationary cycling or overground walking) was provided before testing to minimize variability due to stiffness or fatigue. Physical tests were performed in a designated assessment area to ensure patient safety and standardization of surface and equipment. For all asymmetry tests, both limbs were tested separately with a 1-min rest between trials. Data were entered into a secure digital database immediately following collection to ensure accuracy and traceability.

#### 2.5.1 Sample size calculation

Using G*Power (v3.1.9.7), a sample size of 109 participants was calculated to detect a moderate effect size (f^2^ = 0.15) in a multiple linear regression model with eight predictors, assuming α = 0.05, power = 0.80, and a two-tailed test. To account for potential missing data, a 15% buffer was applied, resulting in a final target sample size of 125 participants.

#### 2.5.2 Data analysis

All statistical analyses were conducted using IBM SPSS Statistics, Version 24.0 (IBM Corp., Armonk, NY). Descriptive statistics (means and standard deviations) were calculated for continuous variables. Between-limb differences in unilateral performance measures (SLSST, Step-Down Test, and mSEBT) were examined using paired-samples *t*-tests. Pearson correlation coefficients were computed to evaluate associations between asymmetry measures and functional performance outcomes (TUG, 30CST) as well as patient-reported outcomes (KOOS-ADL and KOOS–Sport/Rec). To determine independent predictors of outcomes, two multiple linear regression models were constructed. TUG was specified as the dependent variable in Model 1 and KOOS-ADL in Model 2. Predictor variables included demographic factors (age, sex, BMI, time since surgery) and asymmetry measures derived from SLSST, Step-Down Test, and mSEBT. Group differences in functional and patient-reported outcomes between participants classified as having high versus low asymmetry were analyzed using independent-samples *t*-tests. Statistical significance was set at *p* < 0.05 for all analyses. Participants were categorized as having high asymmetry when the inter-limb difference exceeded 15% on any asymmetry measure. This cutoff was based on established conventions in orthopedic and lower-limb functional research, where inter-limb differences greater than 10%–15% are generally considered clinically meaningful and indicative of residual neuromuscular or strength deficits, particularly in post-arthroplasty and knee osteoarthritis populations. Previous studies examining limb loading and strength asymmetries after total knee arthroplasty have applied comparable thresholds to distinguish adaptive variability from clinically relevant imbalance (5, 8). In the absence of universally accepted post-TKA field-test standards, the 15% criterion was selected as a conservative and clinically interpretable benchmark. The final sample size exceeded the minimum requirement established by the *a priori* power analysis, supporting adequate statistical power for regression modeling. Assumptions for parametric testing–including normality, linearity, independence of errors, and absence of multicollinearity–were evaluated and met. Regression diagnostics demonstrated acceptable variance inflation factors and appropriate residual distributions, confirming model stability. Data were screened for completeness prior to analysis, and all results are based on complete-case datasets with clearly defined outcomes and reported confidence intervals to ensure transparency and interpretability.

## 3 Results

Participants (*N* = 125) had a mean age of 68.34 years (SD = 6.12), with a slight female predominance (58.40%), and an average BMI of 28.76 kg/m^2^, indicating an overweight population typical of post-TKA cohorts (Table 1). Functional performance measures reflected mild impairment, with a mean TUG time of 10.43 s and an average of 12.36 repetitions on the 30-Second Chair Stand Test. KOOS scores indicated moderate perceived functional recovery, with mean scores of 71.82 (ADL) and 69.44 (Sport/Rec). Statistically significant asymmetries were observed in all unilateral performance tests, including SLSST (*p* = 0.012), Step-Down Test (*p* = 0.018), and mSEBT anterior reach (*p* = 0.045), with a notable mSEBT asymmetry of 4.33 cm (*p* = 0.030), suggesting persistent side-to-side neuromuscular discrepancies beyond 6 months post-operatively.

**TABLE 1:** Demographic, clinical, and functional characteristics of the sample.

Variable	Mean ± SD or *n* (%)	95% CI	*P*-value
Age (years)	68.34 ± 6.12	67.20–69.48	–
Sex (male/female)	52 (41.60%)/73 (58.40%)	–	–
BMI (kg/m^2^)	28.76 ± 3.45	27.89–29.63	–
Surgical side (right/left)	65 (52.00%)/60 (48.00%)	–	–
Time since surgery (months)	8.23 ± 1.91	7.89–8.57	–
Comorbidity count (mean ± SD)	1.76 ± 1.12	1.52–2.00	–
TUG (seconds)	10.43 ± 1.67	10.11–10.75	–
30-Second Chair Stand Test (reps)	12.36 ± 3.25	11.66–13.06	–
KOOS-ADL (0–100)	71.82 ± 13.58	69.24–74.40	–
KOOS- Sport/Rec (0–100)	69.44 ± 12.79	66.88–72.00	–
SLSST asymmetry (reps)	2.15 ± 1.02	1.92–2.38	0.012
Step-Down Test asymmetry (steps)	3.28 ± 1.27	2.97–3.59	0.018
mSEBT anterior reach (% leg length)	67.12 ± 5.96	65.94–68.30	0.045
mSEBT asymmetry (cm)	4.33 ± 1.51	4.03–4.63	0.030

ADL, activities of daily living; BMI, body mass index; CI, confidence interval; KOOS, Knee injury and Osteoarthritis Outcome Score; mSEBT, modified Star Excursion Balance Test; SD, standard deviation; SLSST, Single-Leg Sit-to-Stand; TUG, Timed Up and Go.

**FIGURE 1:**
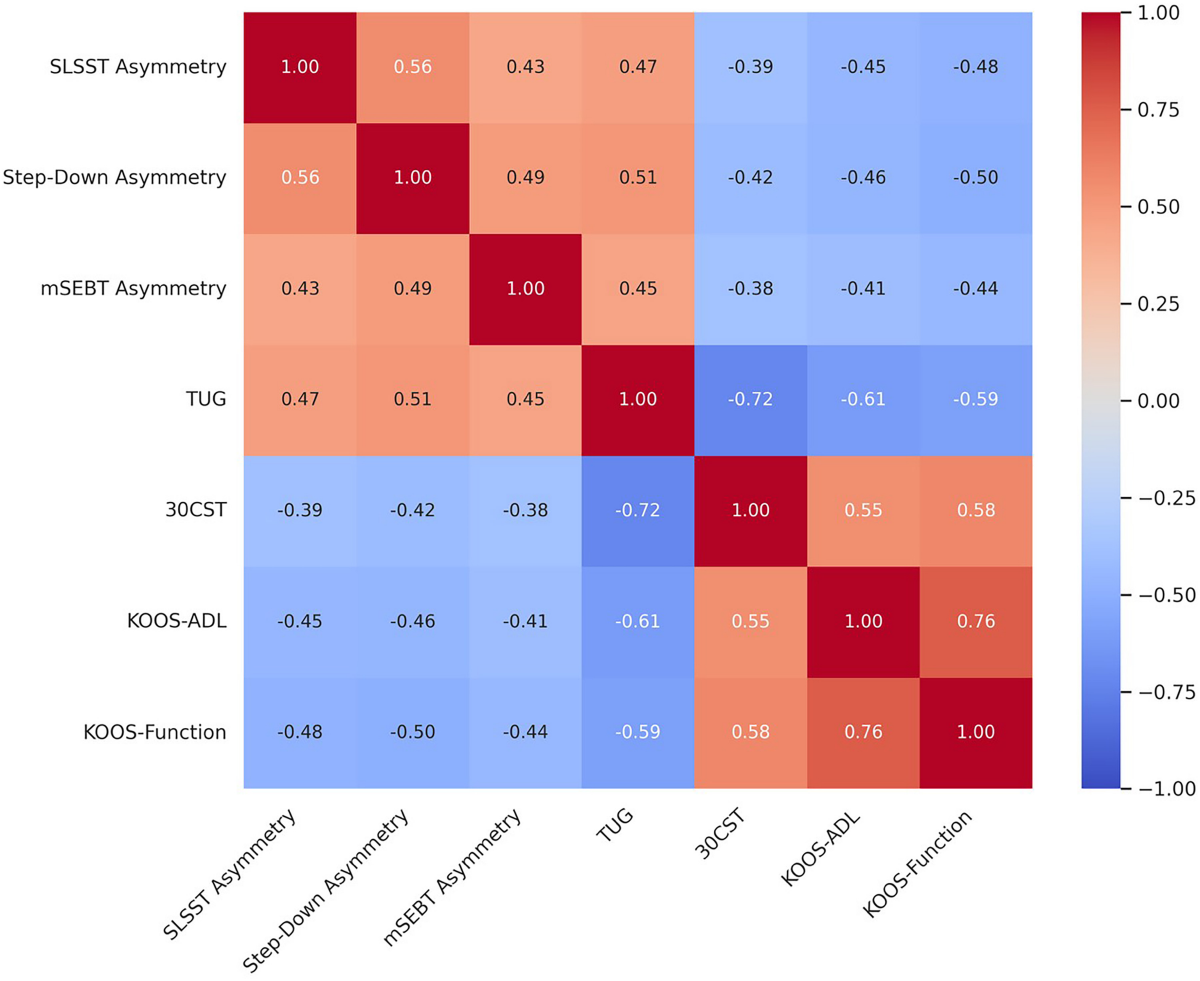
Correlation matrix of asymmetry and sensorimotor measures with functional and self-reported outcomes.

Significant moderate correlations were identified between asymmetry measures and both functional performance and self-reported outcomes, highlighting clinically relevant interrelationships (Figure 1). Greater asymmetry in SLSST, step-down, and mSEBT was consistently associated with poorer performance on TUG (*r* = 0.45–0.51, all *p* < 0.01) and lower 30CST scores (*r* = −0.38 to −0.42, all *p* < 0.02), indicating that increased unilateral deficits relate to slower mobility and reduced strength. Similarly, higher asymmetry correlated negatively with KOOS-ADL and KOOS- Sport/Rec scores (*r* = −0.41 to −0.50, all *p* < 0.01), suggesting that patients with greater side-to-side discrepancies perceive more functional limitations in daily life. Notably, the TUG and 30CST demonstrated strong reciprocal association (*r* = −0.72, *p* < 0.001), while KOOS subscales were highly interrelated (*r* = 0.76, *p* = 0.001), reinforcing their complementary value in assessing post-TKA recovery.

**FIGURE 2:**
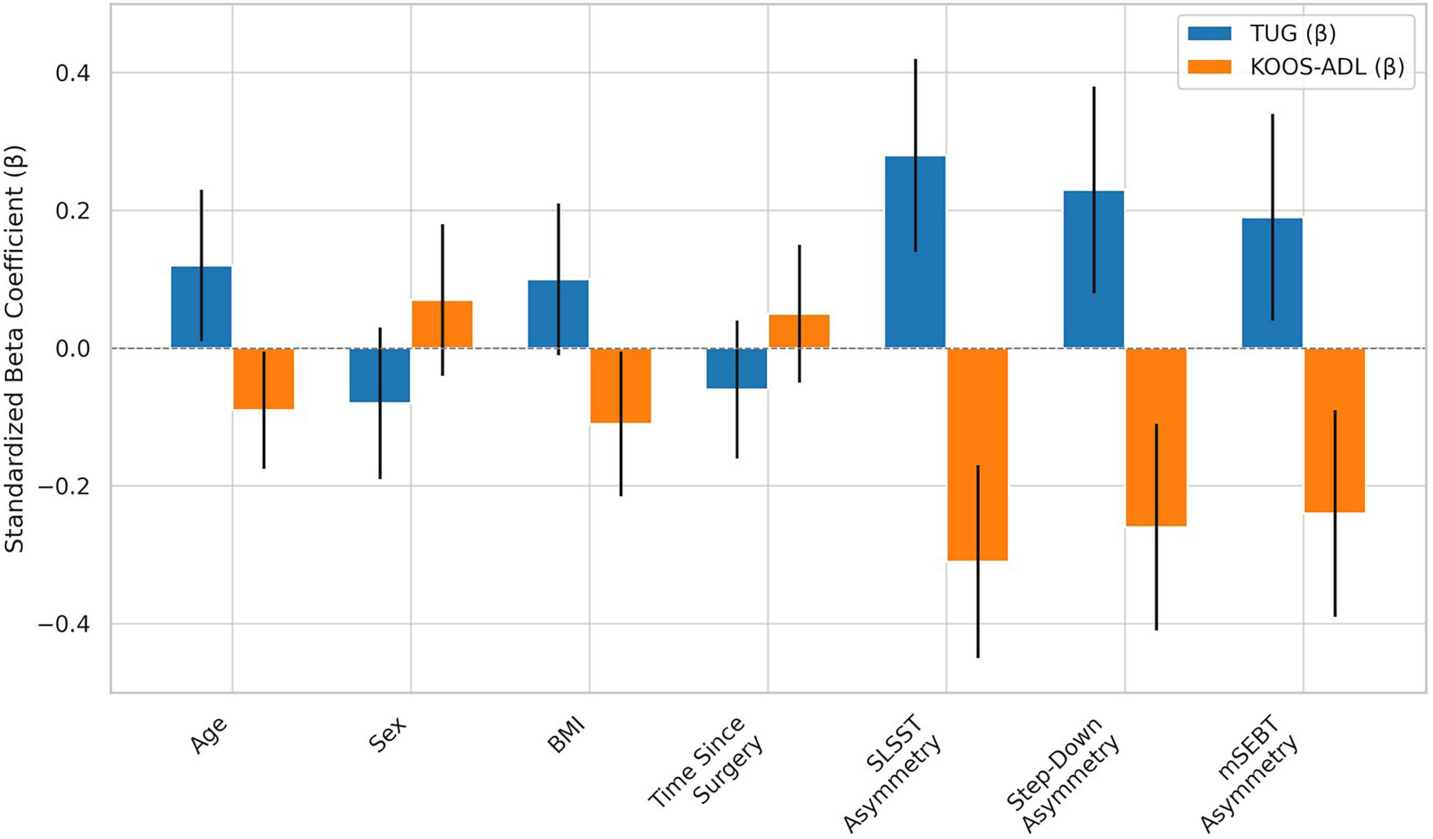
Standardized beta coefficients with 95% confidence intervals for predictors of TUG and KOOS-ADL outcomes.

Both regression models demonstrated significant explanatory power, identifying functional asymmetry and sensorimotor deficits as key predictors of performance-based and self-reported outcomes following TKA (Figure 2 and Table 2). In Model 1, which predicted TUG performance (R^2^ = 0.48, *p* < 0.001), greater asymmetry in SLSST (β = 0.28 [0.14, 0.42]), step-down (β = 0.23 [0.08, 0.38]), and mSEBT anterior reach (β = 0.19 [0.04, 0.34]) were all significantly associated with slower mobility times. In Model 2, explaining KOOS-ADL scores (R^2^ = 0.53, *p* < 0.001), the same variables emerged as significant negative predictors–SLSST asymmetry (β = −0.31 [−0.45, −0.17]), step-down asymmetry (β = −0.26 [−0.41, −0.11]), and mSEBT asymmetry (β = −0.24 [−0.39, −0.09])–indicating that greater unilateral impairments correspond with poorer perceived function in daily activities. Age and BMI were weak but significant covariates in both models, while sex and time since surgery showed no meaningful influence.

**TABLE 2:** Multiple regression models predicting functional (TUG) and self-reported (KOOS-ADL) outcomes.

Predictor variable	Model 1: TUG (β [95% CI])	Model 2: KOOS-ADL (β [95% CI])
Age (years)	0.12 [0.01, 0.23]	−0.09 [−0.18, −0.01]
Sex (Male = 1)	−0.08 [−0.19, 0.03]	0.07 [−0.04, 0.18]
BMI (kg/m^2^)	0.10 [−0.01, 0.21]	−0.11 [−0.22, −0.01]
Time since surgery (months)	−0.06 [−0.16, 0.04]	0.05 [−0.05, 0.15]
SLSST asymmetry (reps)	0.28 [0.14, 0.42]	−0.31 [−0.45, −0.17]
Step-down asymmetry (steps)	0.23 [0.08, 0.38]	−0.26 [−0.41, −0.11]
mSEBT anterior reach asymmetry (cm)	0.19 [0.04, 0.34]	−0.24 [−0.39, −0.09]
Model summary (TUG)		
R^2^	0.48	
Adjusted R^2^	0.45	
F-statistic (df)	15.72 (7, 117)	
Model *p*-value	<0.001	
Model summary (KOOS-ADL)		
R^2^		0.53
Adjusted R^2^		0.50
F-statistic (df)		17.85 (7, 117)
Model *p*-value		<0.001

ADL, activities of daily living; β, standardized beta coefficient; BMI, body mass index; KOOS, Knee injury and Osteoarthritis Outcome Score; mSEBT, modified Star Excursion Balance Test; SLSST, Single-Leg Sit-to-Stand; TUG, Timed Up and Go.

**FIGURE 3:**
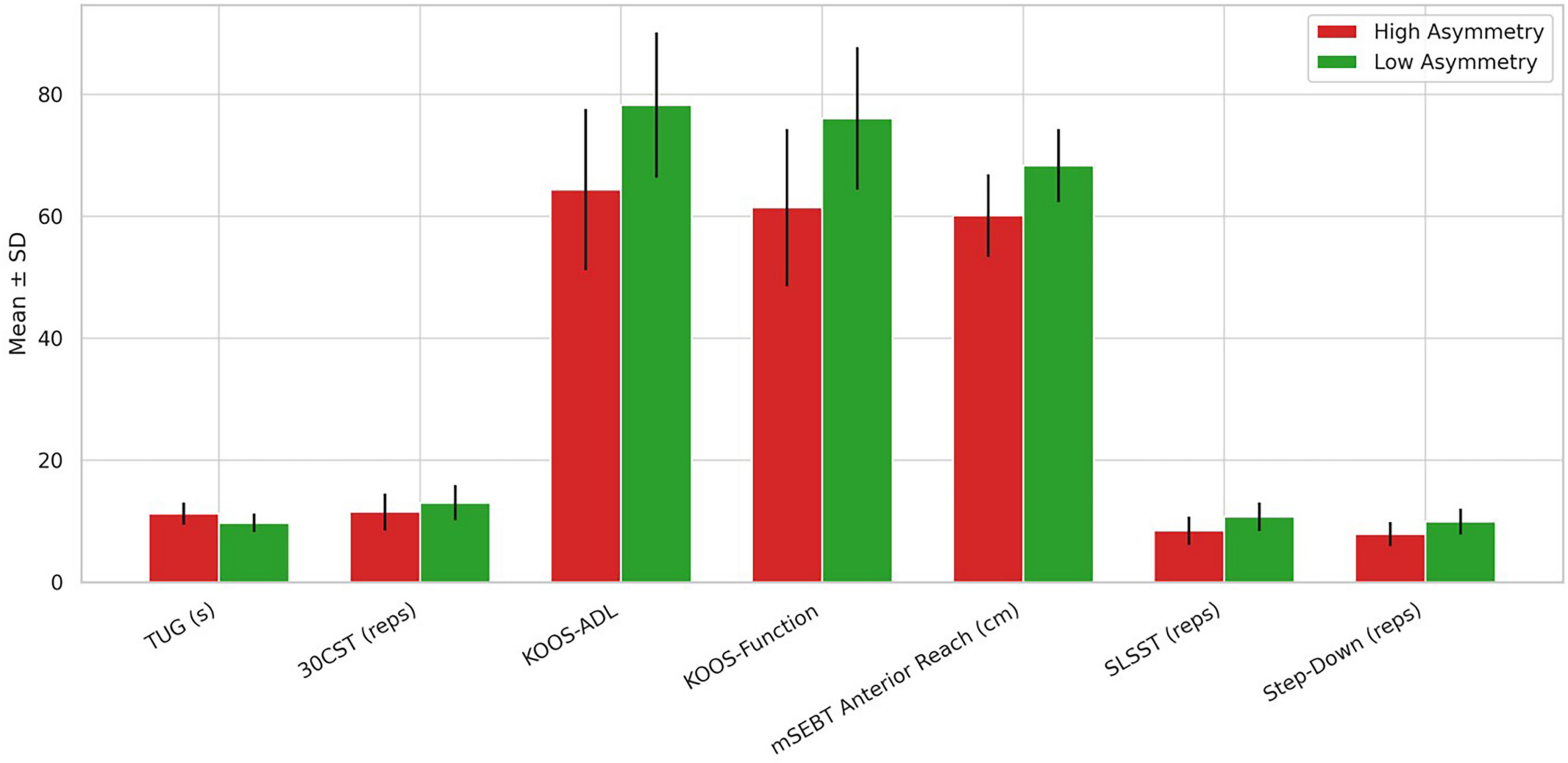
Group differences in functional and PROM outcomes between high and low asymmetry patients post-TKA.

Patients classified in the high asymmetry group exhibited significantly poorer outcomes across all functional and patient-reported measures compared to those in the low asymmetry group, indicating clinically meaningful impairments associated with greater limb imbalance (Figure 3 and Table 3). High asymmetry was linked to slower TUG performance (mean difference = 1.53 s, *p* < 0.001), fewer repetitions on the 30-Second Chair Stand Test (mean difference = −1.50, *p* = 0.006), and substantially lower KOOS-ADL and KOOS- Sport/Rec scores, with differences exceeding 13 points (both *p* < 0.001), reflecting reduced perceived functional ability. Additionally, the high asymmetry group demonstrated significantly reduced mSEBT reach distance and fewer repetitions in both the SLSST and Step-Down Tests, with all differences statistically significant at *p* < 0.001.

**TABLE 3:** Comparison of high vs. low asymmetry groups on functional and PROM outcomes.

Outcome measure	High asymmetry (*n* = 60) Mean ± SD	Low asymmetry (*n* = 65) Mean ± SD	Mean difference [95% CI]	*P*-value
TUG (s)	11.26 ± 1.82	9.73 ± 1.49	1.53 [0.88, 2.18]	<0.001
30-Second Chair Stand Test (reps)	11.52 ± 3.04	13.02 ± 2.89	−1.50 [−2.56, −0.44]	0.006
KOOS-ADL (0–100)	64.38 ± 13.21	78.26 ± 11.92	−13.88 [−18.48, −9.28]	<0.001
KOOS- Sport/Rec (0–100)	61.44 ± 12.88	76.08 ± 11.67	−14.64 [−19.53, −9.75]	<0.001
mSEBT anterior reach (cm)	60.14 ± 6.75	68.34 ± 5.97	−8.20 [−10.61, −5.79]	<0.001
SLSST repetitions (affected limb)	8.43 ± 2.28	10.74 ± 2.35	−2.31 [−3.20, −1.42]	<0.001
Step-Down Test repetitions (affected limb)	7.88 ± 1.97	9.95 ± 2.11	−2.07 [−2.97, −1.17]	<0.001

ADL, activities of daily living; CI, confidence interval; KOOS, Knee injury and Osteoarthritis Outcome Score; mSEBT, modified Star Excursion Balance Test; PROM, patient-reported outcome measure; SD, standard deviation; SLSST, Single-Leg Sit-to-Stand; TUG, Timed Up and Go.

## 4 Discussion

This cross-sectional study aimed to examine the association between functional asymmetry, sensorimotor control deficits, and both objective performance and self-reported outcomes in patients 6–12 months after TKA, using readily available clinical assessment tools. The primary objective was to identify how asymmetry in unilateral functional tasks and dynamic balance measures correlated with standard mobility tests and patient-reported metrics. The secondary objective was to explore whether specific sensorimotor deficits could predict functional mobility and perceived daily living capacity. The results showed consistent, statistically significant associations between greater asymmetry and poorer functional performance, along with lower self-perceived functional ability. Regression modeling further identified key asymmetry variables as significant predictors of both objective and subjective outcomes. Additionally, group comparisons revealed that patients with higher asymmetry levels displayed clinically meaningful impairments across all recovery domains. From a clinical perspective, the identification of persistent post-surgical asymmetry provides actionable information for physiotherapists and physiatrists in tailoring rehabilitation strategies. Targeted interventions may include unilateral strengthening of the operated limb, task-specific eccentric training, neuromuscular re-education, dynamic balance retraining, and progressive limb-loading exercises designed to restore symmetrical movement patterns (27–29). Emphasis on correcting inter-limb discrepancies during functional tasks such as sit-to-stand transitions, stair negotiation, and gait can reduce compensatory overload of the contralateral limb and improve movement efficiency (9). Furthermore, integrating asymmetry-focused assessment into routine follow-up enables early identification of patients at risk of prolonged mobility limitations, falls, or dissatisfaction with surgical outcomes (9, 30). By directly addressing side-to-side deficits rather than relying solely on global performance metrics, clinicians can enhance functional independence, confidence in limb use, and overall health-related quality of life in the mid-to-late postoperative phase following TKA.

Elevated asymmetry in unilateral functional and dynamic balance tasks was significantly associated with diminished performance on standard mobility tests and lower scores on patient-reported outcome measures in individuals 6–12 months after TKA. These findings emphasize the persistence of inter-limb deficits in strength, control, and neuromuscular coordination beyond the early postoperative period, suggesting that biomechanical recovery remains incomplete in many patients (31). Previous studies have consistently documented similar patterns: Yoshida et al. (32) demonstrated that quadriceps strength asymmetry, even months after surgery, was strongly predictive of lower functional performance and slower return to activity. Although some cited work includes longitudinal or mixed-joint arthroplasty samples, the documented relationship between strength asymmetry and functional limitation provides mechanistic support for the present TKA-specific findings, reinforcing the broader relevance of asymmetry as a determinant of postoperative mobility (33). Valpiana et al. (34) and Christensen et al. (35) further reported that gait and load-distribution asymmetries may persist for up to 12 months, resulting in compensatory movement strategies that can undermine mobility. The current results support and extend this line of evidence by showing that basic clinical tools–such as the SLSST, Step-Down Test, and mSEBT –can capture asymmetries that correlate meaningfully with both objective and subjective recovery measures (36). Unlike advanced biomechanical analyses or wearable sensor technologies, these tests are accessible and feasible in most clinical settings, which highlights their utility in outpatient rehabilitation for routine monitoring of functional recovery and discharge decision-making.

Sensorimotor asymmetries were also significant predictors of both performance-based outcomes and self-reported limitations in daily life, as revealed by the multiple regression models (37). In the context of the present study, the term “sensorimotor control” refers to the integrated capacity to coordinate sensory input (proprioceptive and joint position feedback) with appropriate neuromuscular responses to produce controlled, goal-directed movement during functional tasks (37). Although the SLSST, Step-Down Test, and mSEBT inherently reflect elements of muscle strength, endurance, and motor coordination, their performance also depends on dynamic postural regulation, inter-limb load distribution, and real-time modulation of movement patterns (36). Therefore, these measures were conceptualized as composite functional indicators of sensorimotor performance rather than isolated strength tests. This multidimensional interpretation aligns with contemporary rehabilitation models in which strength expression, balance control, and coordinated limb loading are considered interdependent components of functional sensorimotor recovery following TKA (36). Specifically, patients who demonstrated greater side-to-side discrepancies in SLSST, step-down, and anterior reach tasks exhibited poorer mobility on the TUG and reported greater difficulties with daily activities on the KOOS-ADL subscale (38). Importantly, these associations remained significant even after adjusting for age, sex, BMI, and time since surgery, reinforcing the independent contribution of neuromotor control impairments to the overall functional profile of post-TKA individuals. These findings align with the work of Moutzouri et al. (39), who highlighted the predictive value of sensorimotor control deficits–particularly those affecting postural stability and limb loading asymmetry–in determining mobility limitations and fall risk in orthopedic populations. While certain referenced studies examined broader orthopedic or lower-limb arthroplasty cohorts rather than exclusively TKA populations, the principles of sensorimotor integration, unilateral loading behavior, and compensatory movement strategies remain directly applicable to post-TKA rehabilitation, particularly in the context of unilateral joint replacement and asymmetrical recovery trajectories (9). Likewise, Young-Shand et al. (40) observed that impaired neuromuscular control after TKA was associated with altered kinematics and reduced patient-reported confidence in limb use, both of which can adversely affect quality of life. Collectively, the current study adds to this body of knowledge by demonstrating that sensorimotor variables measured through simple, non-instrumented tasks are both sensitive and specific enough to explain significant variance in clinical outcomes (41). This suggests that incorporating such measures into standard post-TKA assessments may help clinicians better identify patients at risk of delayed recovery, enabling more targeted, individualized rehabilitation strategies (42).

### 4.1 Clinical significance

The findings of this study underscore the clinical utility of incorporating simple, low-resource assessments of functional asymmetry and sensorimotor control into routine post-TKA rehabilitation. Standardized tools such as the Single-Leg Sit-to-Stand, Step-Down Test, and modified Star Excursion Balance Test proved effective at detecting persistent unilateral impairments, which were significantly associated with both objective mobility deficits and lower self-reported functional ability. By identifying patients with higher levels of asymmetry using accessible, time-efficient measures, clinicians can tailor rehabilitation programs to target specific deficits, potentially reducing the risk of long-term mobility limitations, compensatory overloading, or dissatisfaction with surgical outcomes. These results highlight the importance of moving beyond bilateral or static functional tests and adopting a more nuanced, side-specific evaluation strategy in outpatient orthopedic settings.

### 4.2 Limitations and areas of future research

This study was limited by its cross-sectional design, which precludes causal inference regarding the observed associations among asymmetry, sensorimotor control, and functional outcomes. Additionally, although the study utilized standardized physical therapy tools suitable for clinical practice, more detailed kinetic or kinematic analyses were not included, which may have offered further insight into the underlying mechanisms of asymmetry. The sample, while representative of a typical outpatient post-TKA population, excluded patients with bilateral TKA, revision surgeries, or advanced contralateral osteoarthritis, which may limit generalizability. In addition, the inclusion criterion requiring independent ambulation without assistive devices likely resulted in a relatively higher-functioning cohort. Consequently, the magnitude of functional asymmetry and sensorimotor deficits observed in this study may underestimate impairments present in individuals who continue to rely on walking aids or who demonstrate greater postoperative disability. Therefore, the current findings should be interpreted with caution when extrapolating to more impaired post-TKA populations, and future research should include a broader functional spectrum to determine whether asymmetry-related deficits are more pronounced in those with limited mobility. Future longitudinal studies should examine the trajectory of asymmetry over time and evaluate whether targeted interventions based on these clinical measures result in superior functional recovery. Additionally, rehabilitation exposure was not systematically quantified or controlled in the present study. Variations in rehabilitation dose, duration, intensity, and type–such as supervised outpatient physiotherapy versus home-based exercise programs–may meaningfully influence the degree of functional asymmetry, neuromuscular recovery, and patient-reported outcomes after TKA. The absence of detailed rehabilitation data represents a potential source of unmeasured confounding, as individuals receiving more structured or intensive therapy may demonstrate different recovery trajectories compared to those with limited or unsupervised rehabilitation. Future investigations should incorporate standardized documentation of rehabilitation characteristics to better delineate their moderating effects on asymmetry and functional performance. Incorporating objective neuromechanical assessments alongside clinical tests may also enhance the precision of rehabilitation planning and prognostication.

## 5 Conclusion

This study demonstrated that greater functional asymmetry and sensorimotor deficits, as measured through simple unilateral tasks and balance tests, are significantly associated with poorer performance and self-reported function in patients 6–12 months after total knee arthroplasty. Asymmetry measures not only correlated with key functional outcomes but also served as independent predictors in regression models, reinforcing their relevance for clinical assessment. The use of accessible, clinically feasible tools enables early identification of at-risk individuals and supports more individualized, asymmetry-targeted rehabilitation approaches in the mid-to-late postoperative phase.

## Data Availability

The original contributions presented in this study are included in this article/Supplementary material, further inquiries can be directed to the corresponding author.
